# miR-135b Plays a Neuroprotective Role by Targeting GSK3β in MPP^+^-Intoxicated SH-SY5Y Cells

**DOI:** 10.1155/2017/5806146

**Published:** 2017-04-17

**Authors:** Jianlei Zhang, Wei Liu, Yabo Wang, Shengnan Zhao, Na Chang

**Affiliations:** Department of Neurology, Huaihe Hospital of Henan University, Kaifeng 475000, China

## Abstract

miR-135a-5p was reported to play a crucial role in the protective effects of hydrogen sulfide against Parkinson's disease (PD) by targeting rho-associated protein kinase 2 (ROCK2). However, the role of another member of miR-135 family (miR-135b) and the underlying mechanism in PD are still unclear. qRT-PCR and western blot showed that miR-135 was downregulated and glycogen synthase kinase 3*β* (GSK3*β*) was upregulated at mRNA and protein levels in MPP^+^-intoxicated SH-SY5Y cells in a dose- and time-dependent manner. MTT, TUNEL, and ELISA assays revealed that miR-135b overexpression significantly promoted cell proliferation and inhibited apoptosis and production of TNF-*α* and IL-1*β* in SH-SY5Y cells in the presence of MPP^+^. Luciferase reporter assay demonstrated that GSK3*β* was a direct target of miR-135b. Moreover, sodium nitroprusside (SNP), a GSK3*β* activator, dramatically reversed the effects of miR-135b upregulation on cell proliferation, apoptosis, and inflammatory cytokine production in MPP^+^-intoxicated SH-SY5Y cells. Taken together, miR-135b exerts a protective role via promotion of proliferation and suppression of apoptosis and neuroinflammation by targeting GSK3*β* in MPP^+^-intoxicated SH-SY5Y cells, providing a potential therapeutic target for the treatment of PD.

## 1. Introduction

Parkinson's disease (PD) is a highly prevalent neurodegenerative disorder in elders, characterized by the gradually progressive and selective loss of dopaminergic (DA) neurons within substantia nigra pars compacta (SNpc), along with the abnormal intracellular protein aggregation of *α*-synuclein (*α*-syn) in Lewy bodies (LBs) [1]. It is estimated that the number of PD patients is expected to double by 2030 [2]. Additionally, loss of DA neurons is generally accompanied by resting tremor, progressive akinesia, and/or postural disturbance [3]. *α*-syn has been shown to activate microglia and subsequently induce production of plenty of inflammatory factors, ultimately leading to neuronal injury [4, 5]. Although previous studies have revealed that various pathological factors such as oxidative stress, mitochondrial dysfunction, protein accumulation, and neurogenic inflammation are implicated in the etiology of PD [6, 7], effective therapeutic strategies for PD patients are still urgently needed.

MicroRNAs are endogenous noncoding RNAs (18–22 nt) that serve as important regulators of gene expression and modulators of inflammatory responses [8]. miRNAs negatively regulate gene expression at pre- and posttranscriptional level by binding to the 3′-untranslated region (3′-UTR) of their target mRNAs [9]. It is reported that miRNAs are essential for the synthesis of neuronal committed progenitors and immature neuron survival and differentiation [10, 11]. Growing evidence from postmortem brain analyses and animal model studies suggests that miRNAs exert a vital role in numerous neuroprotective activities, and dysregulation of these molecules are associated with the early pathogenesis of inflammatory and immune-related neurodegenerative diseases including PD [12, 13]. Thus, it is meaningful to design new therapeutic strategies targeting miRNAs for the treatment of PD. miR-135 family, including miR-135a and miR-135b, is highly conserved among mammals. A previous study reported that miR-135a-5p played a crucial role in the protective effects of hydrogen sulfide against PD by targeting rho-associated protein kinase 2 (ROCK2) [14]. Interestingly, a benzofuran derivative (MBPTA), which was identified as a ROCK inhibitor, could protect against MPP(+)-induced oxidative stress and cell death in SH-SY5Y neuroblastoma cells [15]. However, the role of miR-135b and its underlying mechanism in PD are still unclear.

Glycogen synthase kinase 3 (GSK3) was proved to be involved in numerous cellular processes including cell proliferation, apoptosis, and regulation of different cell signalings [16–18]. As one of the isoforms of GSK-3, GSK3*β* is widely expressed in tissues, especially in the brain [19]. GSK3*β*, an important pathogenic protein kinase for PD, is activated by phosphorylation of Try216 residue located in the kinase domain and inactivated by protein kinase B- (Akt-) mediated phosphorylation of Ser9 residue [20, 21]. GSK3*β* is well-known for its role on glycogen metabolism, activation of transcription factors, and phosphorylation of tau proteins [22, 23]. GSK3*β* plays key roles in the pathogenesis of many neurodegenerative diseases including PD, affecting multiple pathological events encompassing neuroinflammation, neuronal apoptosis, and DA neuron degeneration [24]. It is well documented that GSK3*β* inhibition significantly decreased MPTP-induced neuron injury, ameliorated behavioral impairments caused by MPTP, and has become a therapeutic target for PD [25]. More notably, a previous study reported that GSK3*β* was a target of miR-135 family including miR-135a and miR-135b, which both played an important role in the development of podocyte injury and the disorder of the podocyte cytoskeleton [26]. However, whether miR-135 could target GSK3*β* to exert its biological role in PD remains to be illustrated.

Since neurotoxin 1-methyl-4-phenyl-pyridinium ion (MPP^+^) could elicit a severe PD-like syndrome, we constructed an in vitro model of PD by MPP^+^-induced SH-SY5Y cells. In our study, we explored the role of miR-135b in an in vitro model of PD and whether miR-135b exerted its function in PD by regulating the expression of GSK3*β*.

## 2. Materials and Methods

### 2.1. Cell Culture and Treatment

Human neuroblastoma cell line SH-SY5Y (ATCC, Manassas, VA, USA) was maintained in DMEM (Invitrogen, Grand Island, NY, USA) containing 10% heat-inactivated fetal bovine serum (FBS; Invitrogen) and 1% penicillin-streptomycin at 37°C in a humid incubator with 5% CO_2_. To produce an experimental PD model in vitro, SH-SY5Y cells were exposed to 0.25, 0.5, 1, and 2 mM MPP^+^ (Sigma, St. Louis, MO, USA) for 24 h or treated with 1 mM MPP^+^ for 6 h, 12 h, 24 h, and 48 h.

### 2.2. Cell Transfection

MiR-135b mimics (miR-135b), miR-135b inhibitor, and miRNA negative control (miR-NC) were all purchased from Ambion (Austin, TX, USA). SH-SY5Y cells were plated in a 6-well plate and cultured to reach 80% confluence prior to transfection. Then, SH-SY5Y cells were transfected with miR-135b, miR-135 inhibitor, or miR-NC at the final concentration of 100 nM with Lipofectamine reagent (Invitrogen). At 48 h after transfection, cells were incubated with 1 mM MPP^+^ for 24 h.

### 2.3. Quantitative Real-Time PCR

Total RNA was abstracted from SH-SY5Y cells using TRIzol Reagent (Invitrogen) according to the manufacturer's instructions. For qRT-PCR analyses of GSK3*β* and miR-135b, complementary DNA (cDNA) was synthesized from 100 ng of total RNA using the AMV reverse-transcription system (Promega Corporation, Madison, WI, USA) and Transcriptor First Strand cDNA Synthesis Kit (Roche, Indianapolis, IN, USA), respectively. Expression levels of mRNA were determined with SYBR Green PCR Master Mix (Applied Biosystems, Foster City, CA) on the Applied Biosystems 7500 RT-PCR System (Applied Biosystems). The sequences of specific primers were miR-135b forward, 5′-GCTTATGGCTTTTCATTCCT-3′; reverse, 5′-GTGCAGGGTCCGAGGT-3′; GAPDH forward 5′-ATTCCATGGCACCGTCAAGGCT-3′; and reverse, 5′-TCAGGTCCACCACTGACACGTT-3′. The PCR reaction conditions were as follows: 40 cycles of 95°C for 10 min, 95°C for 15 s, and 60°C for 1 min. The relative expressions of miR-135b and GSK3*β* were quantified by the 2^−ΔΔCt^ method and normalized to GAPDH expression.

### 2.4. Western Blot Analysis

Total protein was extracted from SH-SY5Y cells with a Total Protein Extraction Kit (KeyGen Biotech. Co. Ltd., Nanjing, China). The protein content was detected using Bradford reagent (Bio-Rad, Hercules, CA, USA) by measuring absorbance at 595 nm. Equal amount of the protein (20 *μ*g) was loaded onto 12.5% SDS-PAGE and then transferred to polyvinylidene fluoride (PVDF) membranes. The membranes were first blocked with 5% silk milk at 4°C overnight, then probed overnight with primary antibody anti-GSK3*β* (1 : 500; Cell Signaling Technology, Danvers, MA, USA), anti-cleaved caspase 3 (1 : 500; Cell Signaling Technology), and anti-*β*-actin (1 : 2000; Cell Signaling Technology), and followed by incubating with horseradish peroxidase-conjugated secondary antibody (1 : 2000; Cell Signaling Technology) for 1 h at room temperature. An enhanced chemiluminescence (Pierce, Rockford, IL, USA) and Totallab 2.0 software were applied to visualize and analyze the protein bands. The protein levels were normalized to the internal control *β*-actin expression.

### 2.5. MTT Assay

Cell viability was monitored using the conventional 3-(4,5-dimethylthiazol-2-yl)-2,5-diphenyltetrazolium bromide (MTT) assay. Briefly, SH-SY5Y cells (2 × 10^4^/well) were inoculated into 96-well plates and cultured for 24 h prior to transfection. Cells transfected with miR-NC or miR-135b were treated with 1 mM MPP^+^ for 24 h. Following this, SH-SY5Y cells were treated with 20 *μ*l of MTT solution (5 mg/ml; Sigma) for another 4 h at 37°C. Then the supernatant was discarded gently and 150 *μ*l DMSO was added to each well for 15 min incubation. The absorbance at 490 nm was determined by using a microplate reader (Bio-Rad).

### 2.6. Luciferase Reporter Assay

The entire 3′-UTR fragment of GSK3*β* containing the miR-135b binding sites was amplified and inserted into the downstream of a firefly luciferase reporter gene in the pmirGLO plasmid (Promega Corp., Madison, WI, USA). The sequence of GSK3*β* interacting with the seed region of miR-135b was mutated by a site-directed gene mutagenesis kit (Beyotime Institute of Biotechnology, Beijing, China) and cloned into an equivalent luciferase reporter plasmid. The constructed luciferase reporter plasmids were named as pmirGLO-GSK3*β*-3′-UTR-WT and pmirGLO-GSK3*β*-3′-UTR-MUT, respectively. For luciferase reporter assay, SH-SY5Y cells were cotransfected with 20 ng constructed luciferase reporter plasmids, 10 ng pmirGLO control vectors, and 25 nM miR-135b or miR-NC by Lipofectamine 2000 (Invitrogen). Luciferase activities were measured using a dual luciferase reporter system (Promega) 48 h posttransfection. Renilla luciferase activity was used as the normalization.

### 2.7. Terminal Deoxynucleotidyltransferase-Mediated dUTP Nick-End Labeling (TUNEL) Assay

The in situ cell death detection kit (Roche Molecular Biochemicals, Indianapolis, IN, USA) was used to perform TUNEL assay according to the manufacturer's instructions. Treated SH-SY5Y cells (1 × 10^4^ cells/well) were cultured on four-well glass coverslips, and then, cells were fixed with 4% paraformaldehyde in PBS for 10 minutes at room temperature. The slides were treated with fluorescein-dUTP and TdT in dark at 37°C for 1 h, followed by Hoechst 33342 counterstaining for 30 minutes. The samples were imaged using a laser scanning confocal microscope (Zeiss LSM 510 META, Carl Zeiss, Jena, Germany). The number of TUNEL-positive nuclei cells (blue nuclear stain) was counted in 10 randomly selected fields of a fluorescent microscope in duplicate wells.

### 2.8. Enzyme-Linked Immunosorbent Assay (ELISA)

The treated SH-SY5Y cells from each group were collected and transferred into sterile tubes for further analysis. The concentrations of tumor necrosis factor-*α* (TNF-*α*) and interleukin- (IL-) 1*β* released by SH-SY5Y cells were measured by TNF-*α* and IL-1*β* ELISA kit (IBL, Minneapolis, MN, USA) according to the manufacturer's instructions.

### 2.9. Statistical Analysis

Data were expressed as mean ± standard deviation (SD), and significance was determined at a probability of 5% or less. All data were analyzed by the SPSS13.0 statistical software (IBM, Armonk, New York, USA). Statistical comparisons among multiple groups were carried out by one-way variance (ANOVA).

## 3. Results

### 3.1. miR-135b Was Significantly Downregulated in MPP^+^-Intoxicated SH-SY5Y Cells

**Figure 1:**
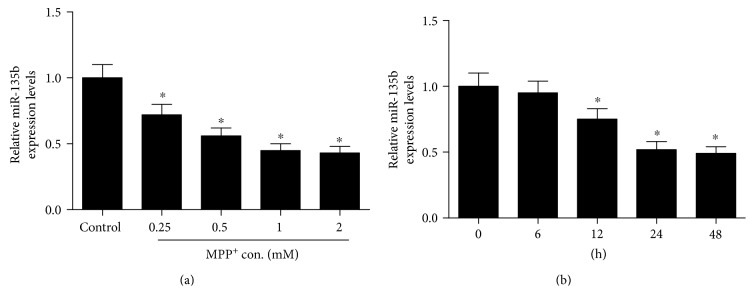
Expression of miR-135b in MPP^+^-intoxicated SH-SY5Y cells. qRT-PCR was performed to examine the expression of miR-135b in SH-SY5Y cells treated with different concentrations (0.25, 0.5, 1, and 2 mM) of MPP^+^ for 24 h (a) or exposed to 1 mM MPP^+^ for different time (6 h, 12 h, 24 h, and 48 h) (b). ^∗^*P* < 0.05 versus control group.

To investigate the role of miR-135b in PD, we first established an in vitro model of PD by treating SH-SY5Y cells with neurotoxin MPP^+^. The expression of miR-135b was detected by qRT-PCR in SH-SY5Y cells treated with different concentrations (0.25, 0.5, 1, and 2 mM) of MPP^+^ for 24 h or 1 mM MPP^+^ at different time (6, 12, 24, and 48 h). The results showed that miR-135b expression was markedly reduced in MPP^+^-intoxicated SH-SY5Y cells in a dose- (Figure 1(a)) and a time-dependent manner (Figure 1(b)).

### 3.2. GSK3*β* Was Dramatically Upregulated in MPP^+^-Intoxicated SH-SY5Y Cells

**Figure 2:**
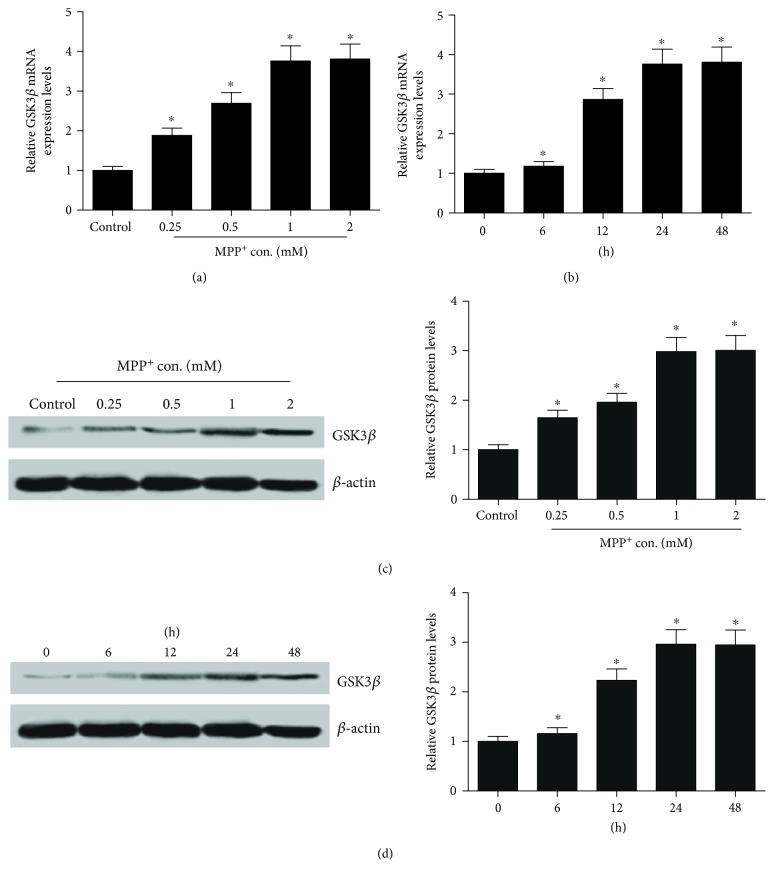
Expression level of GSK3*β* in MPP^+^-intoxicated SH-SY5Y cells. qRT-PCR and western blot were performed to assess the mRNA and protein expression of GSK3*β* in SH-SY5Y cells treated with different concentrations (0.25, 0.5, 1, and 2 mM) of MPP^+^ for 24 h (a and c) or incubated with 1 mM MPP^+^ for different duration (6 h, 12 h, 24 h, and 48 h) (b and d). ^∗^*P* < 0.05 versus control group.

Next, we further explored the expression levels of GSK3*β* in MPP^+^-intoxicated SH-SY5Y cells. Likely, the expression level of GSK3*β* was evaluated by qRT-PCR and western blot in SH-SY5Y cells treated with different concentrations (0.25, 0.5, 1, and 2 mM) of MPP^+^ for 24 h or 1 mM MPP^+^ for different time (6 h, 12 h, 24 h, and 48 h). The results revealed that GSK3*β* was highly expressed in SH-SY5Y cells in a dose- (Figures 2(a) and 2(c)) and a time-dependent manner (Figures 2(b) and 2(d)).

### 3.3. miR-135b Overexpression Attenuated the Effects of MPP^+^ on Proliferation, Apoptosis, and Neuroinflammation of SH-SY5Y Cells

**Figure 3:**
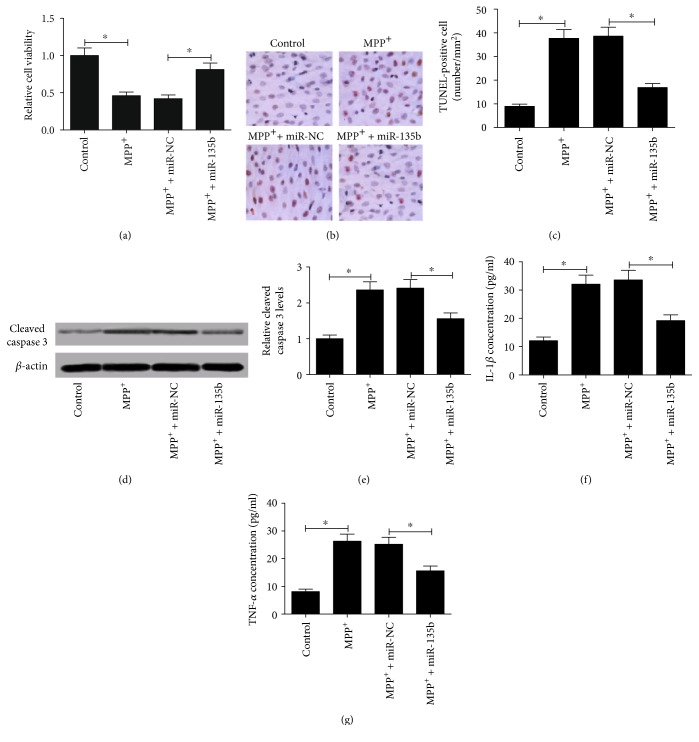
Effect of miR-135b overexpression on viability, apoptosis, and neuroinflammation in MPP^+^-intoxicated SH-SY5Y cells. SH-SY5Y cells were transfected with miR-135b or miR-NC prior to 1 mM MPP^+^ treatment for 24 h. (a) MTT assay was used to determine cell viability in treated SH-SY5Y cells. (b) Apoptosis of treated SH-SY5Y cells was detected by TUNEL assay. (c) Quantification of TUNEL staining was shown. (d) Western blot was performed to evaluate the level of cleaved caspase 3 in treated SH-SY5Y cells. (e) Quantitative analysis of cleaved caspase 3 level. *β*-actin was used as the normalization. The concentrations of IL-1*β* (f) and TNF-*α* (g) in treated SH-SY5Y cells were detected by ELISA. ^∗^*P* < 0.05 versus control group.

To determine the biological function of miR-135b in PD, SH-SY5Y cells were transfected with miR-135b or miR-NC prior to MPP^+^ treatment. As demonstrated by MTT assay, MPP^+^ treatment strikingly inhibited SH-SY5Y cell viability and miR-135b overexpression led to a significant elevation of cell viability in SH-SY5Y cells in the presence of 1 mM MPP^+^ compared with miR-NC group (Figure 3(a)). TUNEL assay was carried out to detect the apoptotic cells in treated SH-SY5Y cells, and the results exhibited that miR-135b transfection conspicuously suppressed MPP^+^-induced apoptosis in SH-SY5Y cells (Figures 3(b) and 3(c)). The neuroprotective role of miR-135b in MPP^+^-induced apoptosis in SH-SY5Y cells was further confirmed by the detection of apoptotic marker cleaved caspase 3. As expected, the level of cleaved caspase 3 was significantly increased in MPP^+^-induced SH-SY5Y cells while miR-135b upregulation attenuated this effect (Figures 3(d) and 3(e)). Furthermore, the effect of miR-135b overexpression on the production of inflammatory cytokines TNF-*α* and IL-1*β* in MPP^+^-intoxicated SH-SY5Y cells was investigated by ELISA. As shown in Figures 3(f) and 3(g), MPP^+^ treatment caused an obvious augment of IL-1*β* and TNF-*α* production in SH-SY5Y cells, whereas enforced expression of miR-135b inhibited MPP^+^-induced TNF-*α* and IL-1*β* secretion. Taken together, these results suggested that the neuroprotective role of miR-135b in PD was confirmed by promoting proliferation, attenuating apoptosis, and suppressing neuroinflammation in MPP*^+^*-intoxicated SH-SY5Y cells.

### 3.4. miR-135b Directly Targeted GSK3*β* and Negatively Regulated Its Expression

**Figure 4:**
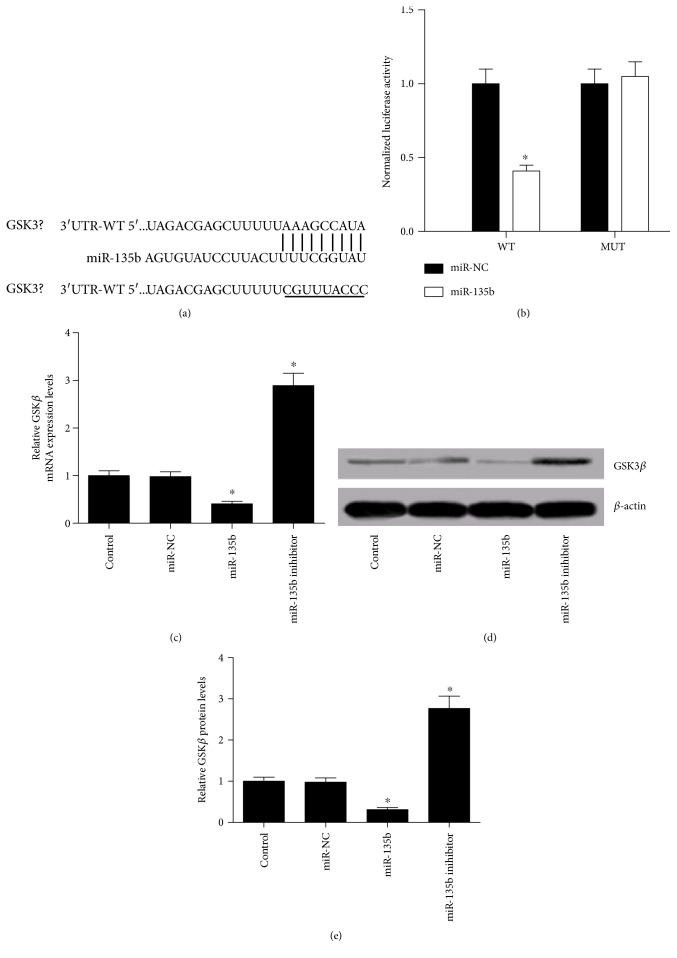
miR-135b directly targeted GSK3*β*. (a) The miR-135b binding sites in the 3′UTR of GSK3*β*. (b) Luciferase reporter assays were carried out in SH-SY5Y cells cotransfected with pmirGLO-GSK3*β*-3′-UTR-WT or pmirGLO-GSK3*β*-3′-UTR-MUT along with miR-135b or miR-NC. (c) qRT-PCR analysis of GSK3*β* mRNA expression in SH-SY5Y cells transfected with miR-135b mimic or inhibitor. (d and e) Western blot analysis of GSK3*β* in SH-SY5Y cells with miR-135b mimic or inhibitor transfection. ^∗^*P* < 0.05 versus control group.

To investigate the underlying mechanism of miR-135b involving in its neuroprotective role in PD, bioinformatics software TargetScan was used to predict the potential target genes of miR-135b. GSK3*β* was found to contain a conserved binding site of miR-135b (Figure 4(a)). To further confirm whether miR-135b could directly target GSK3*β*, a luciferase reporter vector containing wild type or mutant of 3′UTR of GSK3*β* (pmirGLO-GSK3*β*-3′-UTR-WT or pmirGLO-GSK3*β*-3′-UTR-MUT) was cotransfected with miR-135 or miR-NC into SH-SY5Y cells. The analysis of luciferase activities showed that ectopic expression of miR-135b repressed the luciferase activity of pmirGLO-GSK3*β*-3′-UTR-WT compared with miR-NC group. However, this inhibitory effect was abrogated when the seed sequence of miR-135b was mutated in the pmirGLO-GSK3*β*-3′-UTR-MUT vector. To further investigate the interaction between miR-135b and GSK3*β*, the expression of GSK3*β* at mRNA and protein levels was examined in SH-SY5Y cells. The qRT-PCR results revealed that miR-135b overexpression markedly reduced GSK3*β* mRNA (Figure 4(c)) and protein levels (Figures 4(d) and 4(e)), while transfection of miR-135b inhibitor presented the converse effects. These data convincingly demonstrated that miR-135b could specifically target GSK3*β* and regulate its expression.

### 3.5. miR-135b Abated the Effects of MPP^+^ on Proliferation, Apoptosis, and Neuroinflammation by Targeting GSK3*β* in SH-SY5Y Cells

**Figure 5:**
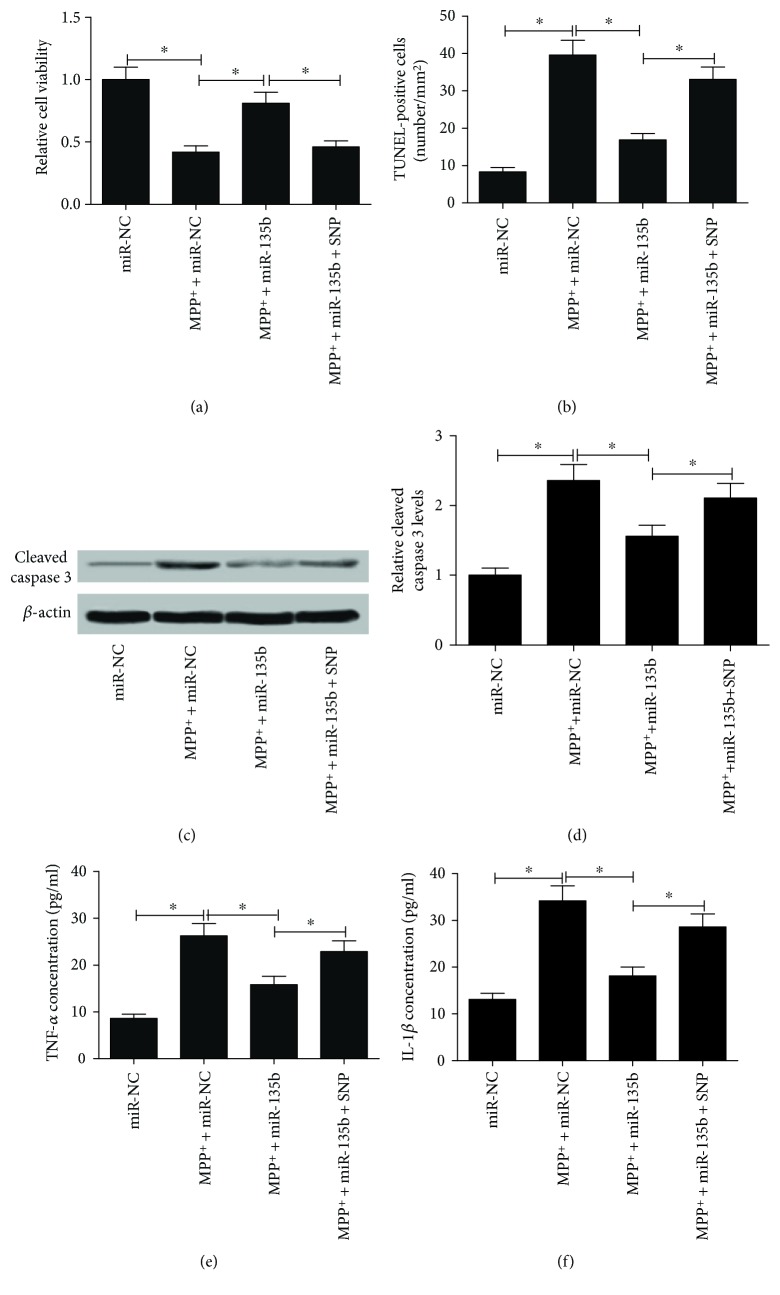
Activation of GSK3*β* reversed the effects of miR-135b on proliferation, apoptosis, and neuroinflammation in MPP^+^-intoxicated SH-SY5Y cells. SH-SY5Y cells with miR-135b transfection were cocultured with 2 mM SNP for 48 h followed by exposing to 1 mM MPP^+^ for 24 h. (a) Cell viability was determined by MTT assay in treated SH-SY5Y cells. (b) Apoptosis of treated SH-SY5Y cells was assessed by TUNEL assay. (c and d) Western blot was carried out to detect the level of cleaved caspase 3 in treated SH-SY5Y cells. The concentrations of TNF-*α* (e) and IL-1*β* (f) in treated SH-SY5Y cells were measured by ELISA. ^∗^*P* < 0.05 versus control group.

GSK3*β* activator sodium nitroprusside (SNP) was used to analyze whether miR-135b exerted the neuroprotective role in PD by targeting GSK3*β*. SH-SY5Y cells transfected with miR-135b were incubated with 2 mM SNP for 48 h and then treated with 1 mM MPP^+^ for 24 h. MTT assay results implied that SNP treatment significantly abolished miR-135b overexpression-mediated cell viability promotion in SH-SY5Y cells in the presence of MPP^+^ (Figure 5(a)). TUNEL assay demonstrated that SNP dramatically overturned miR-135b overexpression-induced apoptosis inhibition in SH-SY5Y cells in the presence of MPP^+^ (Figure 5(b)). Meanwhile, SNP also abrogated the inhibition effect of cleaved caspase 3 induced by miR-135b transfection in MPP^+^-intoxicated SH-SY5Y cells (Figures 5(c) and 5(d)). Moreover, ELISA results implicated that upregulation of miR-135b led to a significant inhibition of TNF-*α* and IL-1*β* production in MPP^+^-intoxicated SH-SY5Y cells while SNP treatment relieved this effect (Figures 5(e) and 5(f)). These finding uncovered that miR-135b improved proliferation, inhibited apoptosis, and reduced neuroinflammation in MPP^+^-intoxicated SH-SY5Y cells by inhibiting GSK3*β*.

## 4. Discussion

In the present study, we examined the role and molecular mechanism of miR-135b in MPP^+^-intoxicated SH-SY5Y cells. MPP^+^-intoxicated SH-SY5Y cells have been widely used as an in vitro model to study DA degeneration of PD [27]. Using the in vitro model of PD, we observed that neuroprotective role of miR-135b overexpression in MPP^+^-intoxicated SH-SY5Y cells was confirmed by a significant increase of cell viability and an obvious inhibition of apoptosis and inflammatory cytokine production. Furthermore, we identified that GSK3*β* was a direct target of miR-135b in SH-SY5Y cells. More importantly, activation of GSK3*β* dramatically impaired miR-135b overexpression-induced neuroprotective effects on MPP^+^-intoxicated SH-SY5Y cells. Therefore, miR-135b overexpression promoted proliferation, attenuated apoptosis, and suppressed neuroinflammation in MPP^+^-intoxicated SH-SY5Y cells by inhibiting GSK3*β*. Our study provides a direct link between miR-135b and GSK3*β* in the progression of PD.

Many scientific findings indicate that dysregulation of some specific miRNAs may be associated with pathogenesis of neurodegenerative diseases including PD [28]. miR-22 overexpression exhibited neuroprotective and reversal effects on a 6-hydroxydopamine- (6-OHDA-) induced cell model of PD by targeting transient receptor potential melastatin 7 (TRPM7) [29]. miR-7 modulated Nod-like receptor protein 3- (NLRP3-) mediated neuroinflammation in the pathogenesis of PD [30]. miR-155 served as a potential therapeutic target for regulating *α*-syn-induced inflammatory responses in models of PD [8]. The present study showed that miR-135b was significantly downregulated in MPP^+^-intoxicated SH-SY5Y cells in a dose- and time-dependent manner. Besides, we noticed that MPP^+^ exposure caused neuron impairments including the reduced cell viability, improved apoptosis, and inflammatory cytokine production while miR-135b overexpression conspicuously relieved these effects, suggesting its neuroprotective role in PD. Similarly, miR-135b was demonstrated to exert a neuroprotective role via direct targeting of *β*-site APP-cleaving enzyme 1 (BACE1) in Alzheimer's disease (AD) [31].

Increasing evidence has emerged to indicate that activated microglia and astrocytes are consistently observed in the SNpc of PD patients and in an animal model of PD [32, 33]. The activated astrocytes contribute to the neuroinflammation process by secreting various proinflammatory molecules such as TNF-*α* and IL-1*β*, triggering the progressive damage of PD [34, 35]. It has been reported that GSK3*β* was a crucial regulator of inflammatory response [36]. A previous study found that inhibition of GSK3*β* by LiCl and SB415286 provided protection against 6-OHDA-induced neuroinflammation in primary cultured astrocytes [37]. Moreover, GSK3 was reported to promote microglial responses to inflammation and promoted inflammation-induced neuronal toxicity [38]. In addition, GSK3*β* is implicated in the regulation of neuronal apoptosis [24]. A previous document clarified that caspase 3 and GSK3*β* activation participated in 6-OHDA-induced DA cell death and the active caspase 3 might be involved in 6-OHDA-induced neuroinflammation of DA neurons [39]. In our study, the data demonstrated the high expression of GSK3*β* in MPP^+^-intoxicated SH-SY5Y cells. Molecular mechanism investigation identified that GSK3*β* was a direct target of miR-135b and miR-135b was revealed to negatively regulate the expression of GSK3*β* in SH-SY5Y cells. In line with the previous study, GSK3*β* was identified as a target gene of miR-135 and miR-135 could regulate GSK3*β* expression in podocytes [26]. GSK3*β* has been previously demonstrated to be a direct target in glioblastoma multiforme [40] and podocyte [26]. Further studies revealed that GSK3*β* activation by SNP could reverse the neuroprotective effects of miR-135 upregulation in MPP^+^-intoxicated SH-SY5Y cells. Thus, we concluded that miR-135b overexpression played a neuroprotective role in PD by inhibiting GSK3*β*.

In conclusion, neuroprotective role of miR-135b overexpression was verified by promoting proliferation, attenuating apoptosis, and suppressing neuroinflammation via targeting GSK3*β* in SH-SY5Y cells, suggesting that miR-135b may act as a potential therapeutic target for the treatment of PD. Further investigations focusing on the downstream signaling pathway of miR-135b/GSK3*β* in the development of PD are needed.
